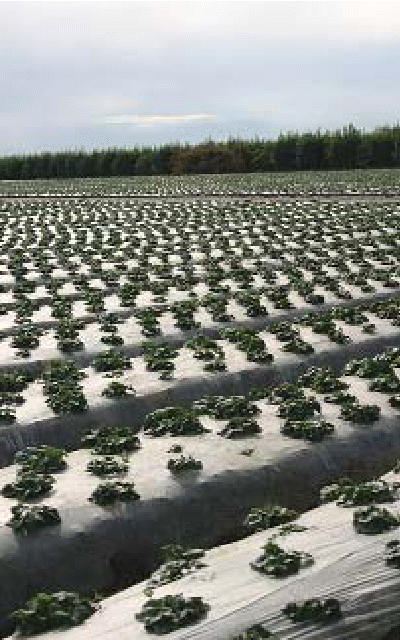# The Beat

**Published:** 2006-09

**Authors:** Erin E. Dooley

## Tracking Toxicants in Canadians

Health Canada announced in May 2006 that it would begin a national biomonitoring program to measure levels of toxic chemicals in the bodies of Canadians. The announcement came as the NGO Environmental Defense prepared to release the results of its own tests, the first look at the amounts of chemicals showing up in Canadian adults and children. That study found 46 of 68 chemicals tested for, with an average of 32 chemicals appearing in adults and 23 in children. Compounds such as polychlorinated biphenyls and DDT were found in children born years after these chemicals were banned. It is not yet known whether the new program, set to begin in late 2007, will be permanent.

**Figure:**
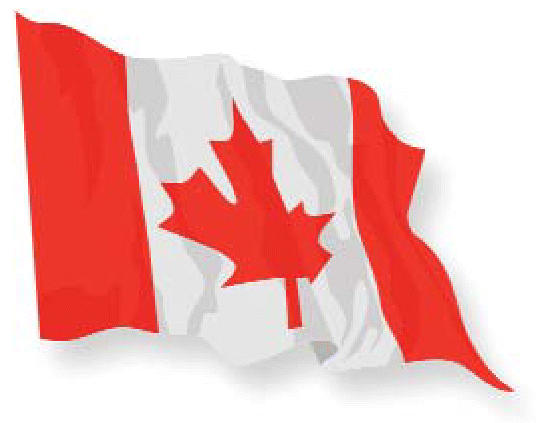


## Citizens Want Free Access to Research Findings

The results of an online Harris poll released 1 June 2006 show that 82% of U.S. adults believe the findings of federally funded research should be available for free online, and that 62% believe free access would lead to quicker discoveries that positively impact health. Heather Joseph, executive director of the Scholarly Publishing and Academic Resources Coalition, commented, “The public recognizes its stake in open sharing of resources, and the Harris data gives voice to their stand.” Senators John Cornyn (R–Texas) and Joseph Lieberman (D–Connecticut) recently introduced the Federal Research Public Access Act of 2006, which would require federal agencies that fund over $100 million in external research per year to make their study results publicly available online.

## Roadside Meth Risk

According to the National Advisory Council on Drug Abuse, every pound of methamphetamine produced means 5 to 7 pounds of toxic materials. Now roadside cleanup volunteers and maintenance workers are being educated about the dangers of picking up litter tossed out when meth labs clean house. People coming across such materials can experience skin burns or lung damage from touching or inhaling fumes from meth waste. Several state and local agencies have created brochures and videos to educate their workers. Hints indicating a roadside meth dump site include bottles with rubber hoses attached, the smell of ammonia, and coffee filters stained red or containing a white powdery residue.

**Figure:**
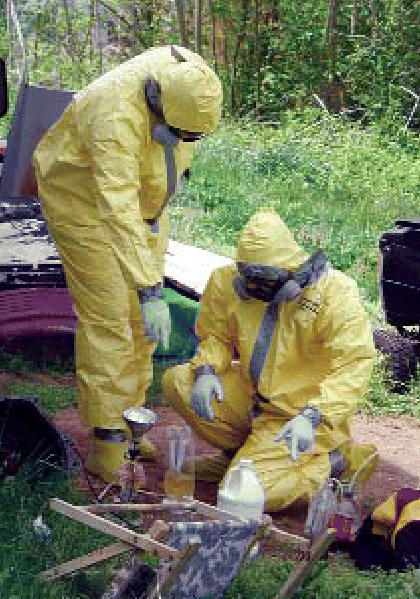


## Cairo Hails New Fleet of Eco-Cabs

Cairo is known for its poor air quality, with its near-permanent haze a mix of industrial emissions, desert sand, and car exhaust fumes. In March 2006, a small fleet of new taxis hit the streets of Egypt’s capital city. Unlike their predecessors, the 150 new yellow Hyundais and Volkswagens feature catalytic converters to filter their exhaust, along with air conditioning and seat belts. A scheme laid out by Egyptian prime minister Ahmed Nazif calls for a total of 1,500 new taxis to be available by the end of 2006. *The Christian Science Monitor* reported on 1 June 2006 that the Egyptian government is also considering fueling the vehicles with natural gas.

**Figure:**
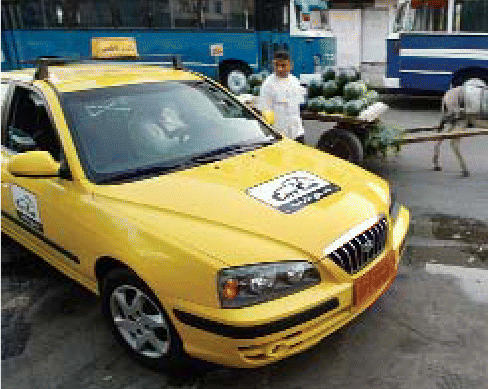


## Living Low Poses Risk

Although only 2.2% of the world’s land area is less than 10 meters above sea level, 10% of the world’s population—some 600 million people—lives at these low elevations. Of these, 60% live in urban areas. A report in the April 2006 news bulletin *Tiempo* by researchers from The Earth Institute at Columbia University states these people are at risk from rising sea levels and increasingly strong storms due to climate change. Geographical location isn’t the only factor that determines vulnerability, however. Although the United States has more urban areas in low-elevation coastal zones than any other country, low-income countries and those designated as Least Developed Countries have fewer resources to rebound from the effects of climate variability.

## A Plan for Farm Plastics

Farmers use “ag plastics” for a wide variety of purposes—dairy and silage bags, coverings for crops, wrappings for hay bales, and more—and thousands of tons are burned, buried, or dumped annually. Now Cornell University researcher Lois Levitan is developing a pilot program to collect and recycle used plastic film sheeting from New York dairies and nurseries.

Levitan reports that about half of discarded ag plastic is burned, which generates emissions of dioxin and other hazardous chemicals. Other waste plastic is often plowed into the ground, where it can become a breeding place for insect pests as well as trap and choke wildlife. Levitan suggests recycling plastics into fence posts, plastic lumber, garbage bags, and other uses, or converting the plastic resin content to a fuel.

**Figure:**